# Bibliometric analysis of racket sport-related injuries: global research landscape, hotspots, and trends

**DOI:** 10.3389/fspor.2026.1897740

**Published:** 2026-09-28

**Authors:** Zongyu Liu, George Liu, Chunying Li

**Affiliations:** 1 School of Health Humanities, Peking University, Beijing, China; 2 School of Basic Medical Sciences, Peking University, Beijing, China; 3 Health Science Library, Peking University, Beijing, China

**Keywords:** bibliometric analysis, epidemiology, global research landscape, racket sports, research hotspot, sport-related injuries

## Abstract

**Objective:**

This study aims to provide a comprehensive overview of racket sport-related injury research, focusing on research progress, collaboration networks, hotspots, and thematic evolution. Drawing on the Web of Science Core Collection, Scopus, and PubMed, the analysis covers 1,837 publications from 76 countries spanning from 2000 to 2025.

**Methods:**

This study employed bibliometric methods combined with statistical analysis. Using CiteSpace 6.4.R1, VOSviewer 1.6.20, SCImago Graphica 1.0.53, and SPSS 26.0, quantitative and visual analyses were conducted on publication trends, geographical distribution, institutional distribution, country/region cooperation networks, core authors, journal distribution, keyword co-occurrences, research hotspots, the evolution of research topics, and highly cited papers. These analyses were supplemented by a correlation analysis, a chi-square goodness-of-fit test, and a cluster analysis.

**Results:**

The global publication volume of racket sport-related injury research exhibited a two-stage growth trend (fluctuating growth and rapid growth), with a significant acceleration after 2020. Geographically, the research was primarily concentrated in Europe, North America, and East Asia, with the United States (527 articles), China (199 articles), and the United Kingdom (152 articles) ranking as the top three in terms of publication quantity. The statistical analysis revealed a significant positive correlation between national 2025 GDP per capita and publication volume (*ρ* = 0.538, *p* < 0.001, 95% CI [0.351, 0.684]). Core research institutions were dominated by sports universities, sports medicine hospitals, and orthopedic research centers, with the Mayo Clinic (USA), University of Liege (Belgium), and Harvard University (USA) as the leading institutions. The international cooperation network was relatively sparse, with merely 15.1% of publications involving cross-national collaboration. The cluster analysis classified research hotspots into 13 core clusters, which were further aggregated into the following six interconnected research domains: epidemiological characteristics and injury prevention, sport-specific injury manifestations, chronic overuse injuries, acute traumatic injuries, imaging-based injury diagnosis, and rehabilitation of sports injuries. The dynamic evolution of research trends was classified into the following three phases: epidemiological investigation and traditional treatment, biomechanical mechanisms and standardized intervention, and intelligent technology and precision prevention. The *Brit J Sport Med* was a core journal source in the field. There were extremely significant differences in article count across three research themes (*χ*^2^ = 354.55, *p* < 0.001, Cramér's V = 0.54), with rehabilitation research differing significantly from both sport injury and injury prevention research.

**Conclusions:**

To move this field forward, scholars should foster transnational interdisciplinary collaboration, expand comparative research across populations and racket sports, implement standardized injury-surveillance and load-management frameworks, and develop generalizable, systematic prevention-rehabilitation systems for racket sport participants.

## 1 Introduction

Racket sports, including tennis, badminton, table tennis, squash, pickleball, and others, are globally popular physical activities widely practiced by both elite athletes and recreational participants across all age groups, offering substantial benefits for cardiovascular function and musculoskeletal health, while also promoting mental wellbeing and social engagement (1). Studies have found that racket sports reduce the risk of all-cause mortality by 47%, making them the most effective type of sport among various physical activities in terms of reducing disease risk. As a result, the number of people participating in racket sports has increased since the COVID-19 pandemic. Skills Framework for the Information Age (SFIA) data shows that racket sports participation as a category grew by approximately 9.4% year-over-year (2, 3). With the growth of sports infrastructure across the globe and the increased awareness of health and fitness, racket sports are immensely attractive as a choice of sport for individuals of all ages. However, racket sports impose unique musculoskeletal loading patterns characterized by repetitive overhead strokes, rapid limb acceleration and deceleration movements, sudden directional changes, and unilateral limb dominance, resulting in a high prevalence of sport-specific acute traumatic and chronic overuse injuries (4). Racket sport-related injury burdens are predominantly driven by cumulative microtrauma rather than violent collision, leading to persistent musculoskeletal disorders that impair athletic performance, reduce physical activity adherence, and induce long-term joint degenerative risks in amateur and professional populations (5). Therefore, it is important to investigate the epidemiology, pathogenesis, treatment, and prevention of injuries associated with racket sports.

Epidemiological studies have identified significant differences in injury characteristics among various racket sports and their participating populations. These studies have shown that acute lower-limb injuries, such as ankle sprains, knee strains, and meniscus injuries, are the dominant match-related trauma, while chronic upper-limb strain injuries, such as rotator cuff injuries, lateral epicondylitis of the humerus, and wrist tendinopathy, are the most common injury types associated with long-term training and daily fitness (6–11). Tennis exhibits the highest overall injury incidence among racket sports, with approximately 48.1 acute injuries per 1,000 match exposures. In contrast, badminton presents a relatively lower but non-negligible injury rate of 3.3 injuries per 1,000 training hours (12, 13). Notably, epidemiological disparities exist between elite and recreational cohorts. Elite athletes suffer from more severe career-terminating injuries with longer time-loss periods, while recreational participants primarily experience mild, recurrent overuse injuries that are frequently overlooked in clinical surveillance (4, 12). Youth and adolescent racket sports participants also exhibit unique injury susceptibility, as their immature musculoskeletal systems make them more vulnerable to overuse damage and growth-related injuries (12, 14). While the epidemiological characteristics of injuries among Western elite athletes are well documented, data on injuries in Asian elite cohorts remain scarce, leading to regional evidence gaps in global assessments of injury burden (5, 15). There is relatively little reporting on independent special studies of the epidemiology of injuries among racket sport athletes, with the majority coming from elite sports investigations at world major events such as the Olympics. For example, in 2024, a retrospective study on 7,809 athletes from Germany, Switzerland, and Austria competing at competitive or recreational levels was conducted. Injury prevalence was highest in team sports (75%), followed by combat (64%) and racket (54%) sports (16). Existing epidemiological studies on racket sport-related injuries predominantly focus on elite athlete populations in Europe and North America. Among these, research on badminton and tennis is the most extensive, followed by squash and pickleball. By comparison, epidemiological data concerning common racket sports such as table tennis are considerably limited, and relevant research reports from Asian countries also remain insufficient. Moreover, some studies have systematically categorized racket sport injuries by disease type and summarized their epidemiological profiles and pathogenic mechanisms. Overall, global large-scale population-based surveys and cohort studies targeting racket sport-related injuries among general and recreational participants are severely lacking, representing a critical research gap in the field of sports injury epidemiology.

Mechanistic research on racket sport-related injuries has advanced substantially over the past two decades, primarily focusing on biomechanical and neuromuscular risk factors. Repetitive asymmetric stroke movements induce abnormal kinetic chain loading, excessive joint torque, and uneven muscle strength balance—core biomechanical mechanisms underlying upper limb overuse injuries (17, 18). Faulty stroke technique, improper racket grip parameters, and inadequate trunk stability further increase local joint stress, accelerating the progression of tendinopathy and soft tissue injury (19, 20). For lower extremity injuries, rapid court movement, insufficient dynamic balance control, and inadequate lower-limb flexibility are the leading mechanical causes of acute sprains and strains (21). Extrinsic factors, including field position, practice conditions, time of season, and training load, also influence the occurrence of injuries (22).

Treatment options for injuries associated with racket sports include physiotherapy, medication, and surgery. Physiotherapy is primarily used for chronic cumulative injuries, such as shockwave therapy, acupuncture, and massage (23–25). Medication is mainly used to control inflammation and pain in acute injuries (26) and surgery is primarily used for the emergency management of acute injuries (27). In recent years, the emergence of AI technology and wearable devices has led to a significant shift in approaches to the prevention and control of racket sport-related injuries (28, 29). Traditional, one-size-fits-all intervention models are gradually being replaced by precise, personalized prevention strategies, specifically, training corrections, fitness interventions, and equipment adjustments tailored to an individual's biomechanical deficiencies, variations in physical fitness, and injury history. Standardized, one-size-fits-all prevention and control programs fail to address the diverse injury risks among populations of different ages, skill levels, and sports disciplines, resulting in limited actual effectiveness. In contrast, personalized injury prevention focuses on an individual's modifiable risk factors to enable the precise implementation of risk screening, technical corrections, and tiered interventions. It has thus become the core direction for the future of injury prevention and control in racket sports (30, 31).

Although there have been many achievements in research on the epidemiology, injury mechanisms, risk factors, diagnosis, treatment, and prevention of racket sport-related injuries, the overall evolutionary trajectory, research hotspots, structural deficiencies, and translational gaps of this field remain unsystematically summarized. Previous reviews have mostly focused on single sports or specific injury types, lacking a comprehensive summary of global research progress on racket sports injuries. Bibliometric analysis, an effective tool for quantitative evaluation of academic research, can objectively reflect the development context, core forces, and evolution trends of a research field by systematically processing and analyzing literature metadata. Statistical methods further deepen the interpretation of results, revealing the intrinsic relationships between research output and influencing factors. Although previous bibliometric studies have focused on specific injuries (e.g., tennis elbow, tennis leg, and tendon and ligament injuries) or broad fields (e.g., Summer and Winter Olympic sports research) (32–37), there is a lack of systematic bibliometric analysis covering all racket sports and their related body injuries. Therefore, this study used bibliometric methods to systematically analyze the publication trends, geographical and institutional distribution, cooperative networks, research hotspots, and emerging frontiers of global racket sports injury research. The findings aim to summarize existing research; explore unresolved scientific questions; and provide comprehensive insights for future epidemiological investigations, mechanistic studies, and personalized prevention research on racket sport-related injuries.

## 2 Materials and methods

### 2.1 Data sources and search strategy

The specialized databases in sport sciences, sport medicine, and injury rehabilitation mainly include SPORTDiscus, Web of Science Core Collection (WOSCC), Scopus, and PubMed. SPORTDiscus is the optimal database for basic research in sports science, physical education, and sports practice. However, due to the restriction of not having ordering permissions, this database could not be used. PubMed has strong coverage of the literature on clinical medicine, sports injuries, and rehabilitation. WOSCC has strong coverage of core journal articles on sports science and sports injuries. Scopus contains a slightly larger volume of literature on sports science, sports injuries, and rehabilitation than WOSCC; if only PubMed and WOSCC were used, studies on sports science would be omitted. Therefore, the latter three databases were selected to retrieve studies for analysis. Data retrieval was completed on 22 January 2026. The data range was set from 1 January 2000 to 31 December 2025, the study type was set to review and journal articles, and the language was set to English. The search strategy was as follows:

- WOSCC: TS = (“racquet sports” OR “racket sports” OR “padel sports” OR “badminton” OR “tennis” OR “tabletennis” OR “table tennis” OR “pickleball” OR “squash ball” OR “squash sports”) AND TS = (“injury” OR “injuries”) NOT TS = (pumpkin OR vegetable)
- Scopus: TITLE((“racquet sports” OR “racket sports” OR “padel sports” OR “badminton” OR “tennis” OR “tabletennis” OR “table tennis” OR “pickleball” OR “squash ball” OR “squash sports”) AND NOT (pumpkin OR vegetable OR cucurbita*) AND (“injury” OR “injuries”)) OR KEY((“racquet sports” OR “padel sports” OR “racket sports” OR “badminton” OR “tennis” OR “tabletennis” OR “table tennis” OR “pickleball” OR “squash ball” OR “squash sports”) AND NOT (pumpkin OR vegetable) AND (“injury” OR “injuries”))
- PubMed: (“Racquet Sports”[MeSH Terms] OR “racket sports”[tiab] OR “padel”[tiab] OR “badminton”[tiab] OR “tennis”[MeSH Terms] OR “tennis”[tiab] OR “tabletennis”[tiab] OR “table tennis”[tiab] OR “pickleball”[tiab] OR “squash ball”[tiab]) AND (“Athletic Injuries”[Mesh] OR “injury”[All Fields] OR “injuries”[All Fields]) OR (“Racquet Sports/injuries”[Mesh])

### 2.2 Literature screening

The literature screening process adhered to the Preferred Reporting Items for Systematic reviews and Meta-Analyses (PRISMA) guideline (38), as illustrated in Figure 1. After data export from the three databases, duplicate records were removed using NoteExpress v4.0. Priority was given to records from WOSCC due to its comprehensive metadata, followed by Scopus and PubMed. The criteria for screening irrelevant literature were as follows:

**Figure 1:**
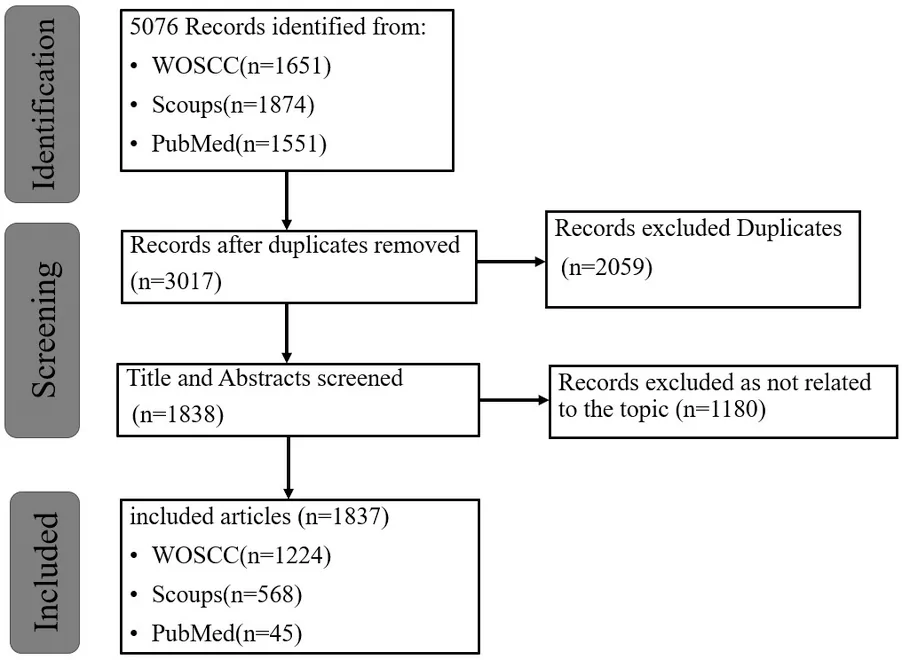
PRISMA screening flowchart.

**Inclusion criteria**: Peer-reviewed journal articles, review articles, and conference proceedings with clear research themes and complete metadata; studies focusing on racket sport-related injuries (including epidemiology, mechanism, risk factors, diagnosis, treatment, and prevention).

**Exclusion criteria**: Retracted publications, book reviews, editorial comments, meeting abstracts, and case reports with insufficient sample size; duplicate studies; studies irrelevant to racket sports or bodily injury.

The initial search yielded 5,076 records: 1,651 from WOSCC, 1,874 from Scopus, and 1,551 from PubMed. After removing 2,059 duplicate records, 3,017 records remained for manual screening. Finally, 1,837 records were included.

### 2.3 Data cleaning

The purpose of data cleaning is to identify and correct errors and inconsistencies in the original dataset to improve the data quality and ensure the accuracy and consistency of the data. The data cleaning principles were as follows:

**Standardizing terminology**: The names of countries, institutions, and authors were standardized to ensure consistency. For instance, “United States” and “USA” were unified as “USA.” “Turkey” and “Turkiye” were consolidated into “Turkey.” “England,” “Scotland,” “Wales,” and “UK” were consolidated into the “United Kingdom.” “CNRS – National Institute for Biology (INSB),” “CNRS – Institute for Information Sciences & Technologies (INS2I),” and “CNRS – Institute for Engineering & Systems Sciences (INSIS)” were unified as “Centre National de la Recherche Scientifique (CNRS).” Variations of authors’ names were unified, with “Ellenbecker, Todd S.,” “Ellenbecker, T.S.,” and “Ellenbecker, Todd” unified as “Ellenbecker, Todd S.,” for example.

**Unified specification of synonyms.** For instance, “racket sports” and “racquet sports” were unified as “racket sports.” “Epidemiologic studies” and “epidemiological studies” were standardized into “epidemiology.” “ACL” and “anterior cruciate ligament” were consolidated into “anterior cruciate ligament.”

**Supplementary information.** Vocabulary with incomplete information was supplemented. For instance, “imaging” was changed to “imaging technology.” “Zagreb” was changed to “Zagreb, Croatia.” “Indiana” was changed to “Indiana, USA.”

### 2.4 Research methods

#### 2.4.1 Bibliometric analysis

Excel 2021 was used to sort and count basic literature information (publication year, annual citations, country/region, author, institution, journal, and keywords). This study examined the temporal distribution of annual publications and computed yearly citation metrics.

There are two categories of indicators to assess academic impact. The first category consists of absolute quantitative indicators, such as annual citation counts, which measure the total number of citations accumulated by all retrieved publications in a given year (39). The second category consists of relative quantitative indicators, such as the average annual citations per paper, which is used to measure the impact of publications within a specific field and helps compare research influence across different fields by normalizing disparities in citation density (40). Citations per paper were utilized in this study to quantify academic impact. This indicator can compare the annual trend of overall academic influence in racket sport-related injuries.

Core author group and core institutions group were identified based on Lotka's Law and Price's Law. Lotka's Law describes the distribution of research output among authors in a scholarly field: the number of authors publishing n papers is approximately inversely proportional to 1/n^2^ (41). Lotka's Law states that high-yield authors are distributed among a small group of people, which is the core author group we need to target. Price's Law posits that half of all published papers are produced by the square root of the total number of authors in a research field (42). The core author group was selected in racket sport-related injuries research according to the core author formula of Price's Law, i.e., core author publication volume is ≥0.749  ×  √Nmax, where Nmax = maximum publication volume of a single author.

#### 2.4.2 Visualization analysis

CiteSpace 6.4.R1 was used to illustrate the keyword clustering analysis by calculating a keyword similarity matrix to form research clusters, which were labeled and interpreted based on core keywords, keyword cluster maps, keyword timeline maps, time zone maps, and keyword burst maps to visualize academic connections and research evolution. VOSviewer 1.6.20 was used to illustrate institution and author cooperation networks. SCImago Graphica1.0.53 is a free graphing software that supports GML data formats and was used to finish the country/region cooperation map.

#### 2.4.3 Statistical analysis

SPSS 26.0 was used for statistical testing, namely, analysis of the compound annual growth rate (CAGR) of published papers. Pearson’s correlation coefficient was used to explore the correlation between national gross domestic product (GDP) per capita and publication volume. A linear regression model was established to identify key influencing factors of research output.

The growth of the number of published papers is calculated using CAGR and was estimated via exponential fitting. The exponential model $y_{t}=ae^{bt}$ was linearized through natural logarithm transformation as lny=lna+bt, and the continuous growth coefficient *b* was acquired via ordinary least squares linear regression. The CAGR value was calculated by the formula, $CAGR=e^{b}-1$, where *b* refers to the slope generated by regressing log-transformed indicators on publication years (43). The corresponding coefficient of determination *R^2^* was calculated as $R^{2}=1-SS\mathrm{res}/SStot$ to quantify the share of variance in indicators explained by time, so as to evaluate the stability and reliability of growth trends for the two stages of 2000–2019 and 2020–2025.

**Correlation analysis and model validation.** A simple and widely adopted bivariate statistical approach combining Pearson’s correlation analysis and ordinary least squares (OLS) simple linear regression was utilized to systematically investigate the relationship between national research output on racket sport-related injuries and macroeconomic capacity. Specifically, national publication volume was set as the dependent variable and 2025 GDP per capita (USD) (44) was defined as the independent variable, based on valid data from 73 countries and regions. Given the pronounced right-skewness of both variables (Shapiro–Wilk test, *p* < 0.001 for both), we employed the following three complementary correlation estimators: Pearson's product-moment correlation coefficient on raw (45) and log_₁₀_-transformed data, Spearman's rank-order correlation (46), and Kendall's tau-b. Moreover, 95% confidence intervals (CIs) for the Pearson’s coefficients were derived via Fisher's z-transformation, whereas rank-based CIs were obtained using the Fieller approximation on transformed ranks. We further fitted the following two ordinary least-squares (OLS) regression models: (i) a raw linear model regressing publication volume on GDP per capita, and (ii) a double-log (log–log) model in which both variables were log_₁₀_-transformed, enabling the slope coefficient to be interpreted as an elasticity—the percentage change in publication volume associated with a 1% change in GDP per capita. Model adequacy was assessed using the coefficient of determination (*R^2^*), the omnibus *F*-test, and a battery of diagnostic procedures as follows: the Shapiro–Wilk and Anderson–Darling tests for residual normality, the Breusch–Pagan test for heteroscedasticity, the Durbin–Watson statistic for autocorrelation, and Cook's distance for influential observations. A robustness check was conducted by re-estimating all correlations after excluding the United States, the most influential outlier.

Analysis of JCR-classified journal data was performed in Python (SciPy) using the chi-square goodness-of-fit test, pairwise comparisons with the Bonferroni correction, and Cramér's V for effect size. The chi-square goodness-of-fit test was conducted to determine whether the distribution of articles across the three categories (Rehabilitation, Sport Injury, and Injury Prevention) deviated significantly from a uniform distribution (47). *Post hoc* pairwise chi-square tests were conducted with the Bonferroni correction (adjusted *α* = 0.0167) to identify which specific categories differed (48). A larger effect size was used to verify whether there were differences in the number of articles across the three categories (49).

#### 2.4.4 Statistical criteria

##### 2.4.4.1 Criteria for countries and regions

If multiple authors of a single publication belonged to the same country or region, that country/region was only counted once. No differentiation was made regarding the relative contribution of each participating country within one publication.

##### 2.4.4.2 Criteria for institutions

If multiple authors of a single publication were affiliated with the same institution, that institution was only counted once. No differentiation was made regarding the relative contribution of each participating institution within one publication.

##### 2.4.4.3 Criteria for Authors

Each author listed on a publication was counted once, regardless of their specific roles in the manuscript. Author contributions, including first authorship and corresponding authorship, were not differentiated in the statistical analysis. If an author was affiliated with two institutions in two different countries, each country and each institution was counted once.

International cooperation refers to collaborative research conducted by authors from two different countries, with each country counted once. Research collaboration among authors from different institutions within the same country was considered domestic cooperation, and the country of the authors was counted only once.

## 3 Results

### 3.1 Quantitative description

After automatic deduplication processing and manual screening, a total of 1,837 publications were included for the analysis, including 216 reviews and 1,621 journal articles, along with 8 conference papers. These publications involve contributions from 76 countries and regions, 1,164 research institutions and 6,716 authors, covering 571 published journals and 3,097 distinct keywords. The dataset contains 53,567 cited references, and the total number of citations accumulated by all retrieved documents is 42,555.

### 3.2 Temporal distribution of annual publication and annual citation per paper

The number of global publications on racket sport-related injuries presents a prominent two-phase growth trend (2000–2025), as illustrated in Figure 2.

**Figure 2:**
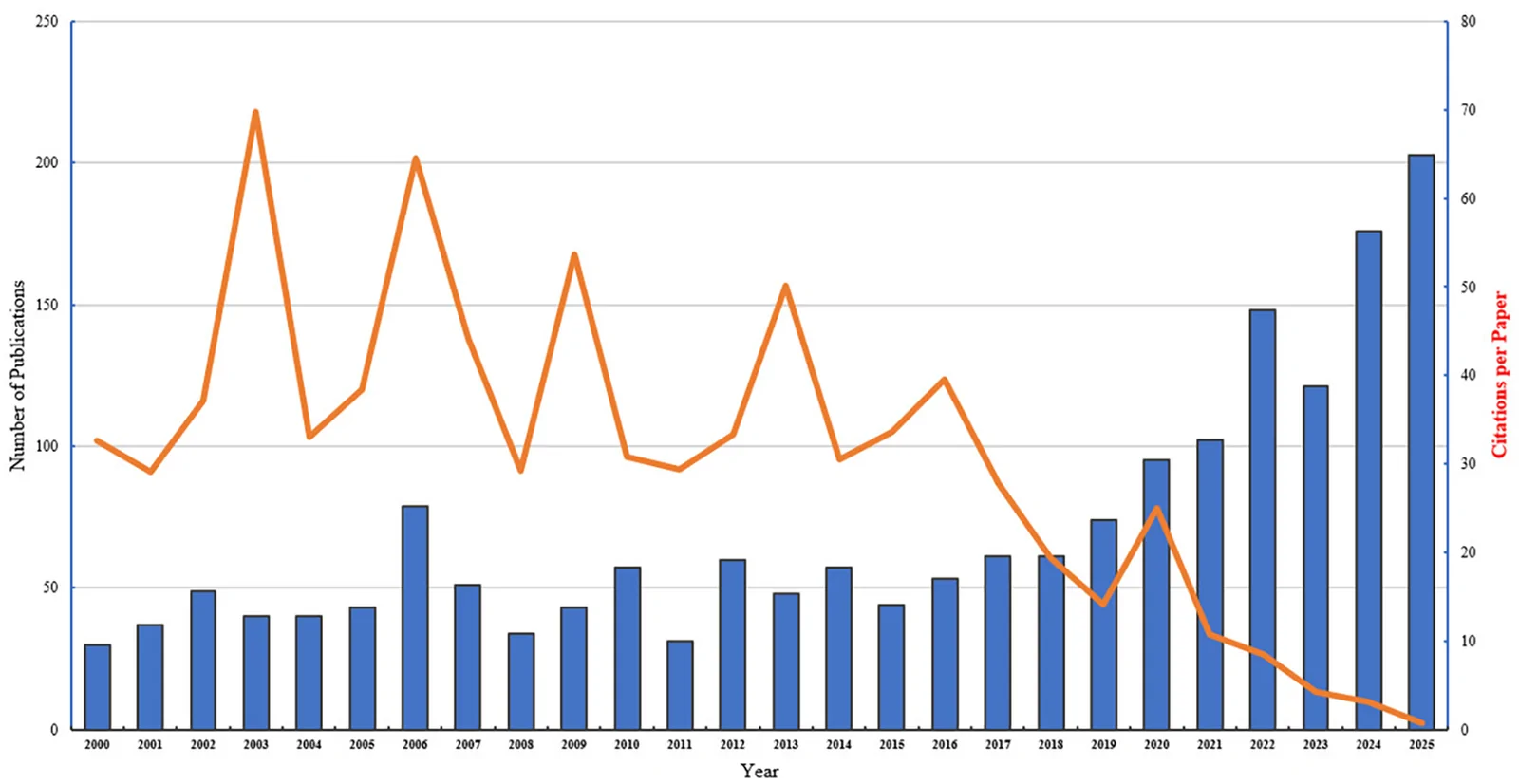
Annual publications and citations per article on racket sport-related injuries (2000−2025).

**Fluctuating growth period (2000–2019)**: The annual publication volume grew from 30 to 74 with marked inter-annual fluctuations. Notable high-growth years included 2006 (+83.72%) and 2012 (+93.55%), while negative-growth periods occurred in 2007–2008, 2011, 2013, and 2015. The CAGR for this period was 2.55%, with an *R^2^* of 0.283. Despite this exponential-fitted annual growth rate over the 20-year span, the low *R*^2^ indicated that time explained merely 28.3% of the variance in log-transformed publication output. Overall, publications exhibited slow and highly variable growth, with temporal factors contributing modestly to annual output changes.

**Rapid growth period (2020–2025)**: The annual publication volume rose from 95 in 2020 to 203 in 2025, showing an overall upward trend. The CAGR increased markedly to 16.12%, accompanied by an *R*^2^ of 0.843. This value represented the average annual exponential growth rate over the 6-year period; the high *R*^2^ indicated that time could explain 84.3% of the variance in log-transformed publication volume. Accordingly, publication output exhibited rapid and relatively stable growth, with the publication year exerting a strong explanatory effect on changes in publication counts. Compared with the 2000–2019 fluctuating-growth phase, this period exhibited a far higher growth rate and larger publication rate increase, alongside much more stable inter-annual growth.

**Annual citations per paper (2000–2025):** The statistical analysis showed that 1,492 articles had been cited, accounting for 81.2% of the 1,837 articles. There is an inverse relationship between the number of papers published and the average number of citations per paper, i.e., while the number of papers published has continued to rise (with the growth rate accelerating particularly since 2020), the average number of citations per paper has been declining year on year. This decline is primarily due to a delay in the citation time window—papers published in recent years have not yet had sufficient time to accumulate citations—and does not reflect a decline in the academic quality of individual papers.

### 3.3 Geographical and institutional distribution

#### 3.3.1 Geographical distribution and correlation analysis

The top 10 countries in terms of publication volume and their GDPs are shown in Table 1. Given substantial right skewness in publication volume and GDP per capita across countries, Spearman's rank-order correlation and double-logarithmic OLS regression were used for primary inference. Spearman's *ρ* revealed a moderate positive monotonic association between national publication output and GDP per capita (*ρ* = 0.538, *p* < 0.001). The log₁₀-log₁₀ regression yielded a statistically significant elasticity estimate: a 1% increase in GDP per capita was associated with a 0.652% increase in publication volume [log₁₀(Publication)=*β*_0_+*β*_1_ log_₁₀_ (GDP per capita) +*ε*, *ρ*=0.652, 95% CI: 0.400–0.904, *p* < 0.001, *R*^2^ = 0.273]. Residual diagnostics supported model adequacy, and the robustness analysis excluding the United States confirmed the stability of these estimates. This result demonstrates that regions with superior economic development generally generate more academic output on racket sports injuries.

**Table 1:** Top 10 countries by number of publications on racket sport-related injuries (2000–2025).

Rank	Country	Number of publications	Proportion (%)	GDP per capita (USD, 2025)
1	USA	527	22.5	90,026.5
2	China	199	8.5	13,862.0
3	United Kingdom	152	6.5	57,602.9
4	Spain	125	5.3	38,627.2
5	Australia	124	5.3	65,129.7
6	France	89	3.8	48,985.7
7	Germany	88	3.8	60,496.4
8	Japan	73	3.1	35,951.0
9	Canada	64	2.7	55,697.7
10	Italy	63	2.7	43,308.6

GDP per capita data is from the World Bank 2025 report (44).

Eight country collaborative networks were found in the literature on racket sport-related injuries (Figure 3A). In cluster 1, India was shown to have close cooperation with countries such as Qatar, Malaysia, and Turkey. In cluster 2, France was shown to have close cooperation with countries such as Japan, Greece, and Russia, while in cluster 3, Canada was shown to have close cooperation with countries such as Denmark, Sweden, and Switzerland. Furthermore, in cluster 4, Italy was shown to have close cooperation with countries such as Poland, Croatia, and Slovenia, and in cluster 5, Germany was shown to have close cooperation with countries such as Brazil, Chile, and Belgium. In cluster 6, China was shown to have close cooperation with countries and regions such as Taiwan, Hungary, New Zealand, and South Korea, while in cluster 7, the USA was shown to have close cooperation with countries such as the United Kingdom, Singapore, and Australia. Finally, in cluster 8, Spain was shown to have close cooperation with countries such as Mexico, Angola, and Iraq.

**Figure 3:**
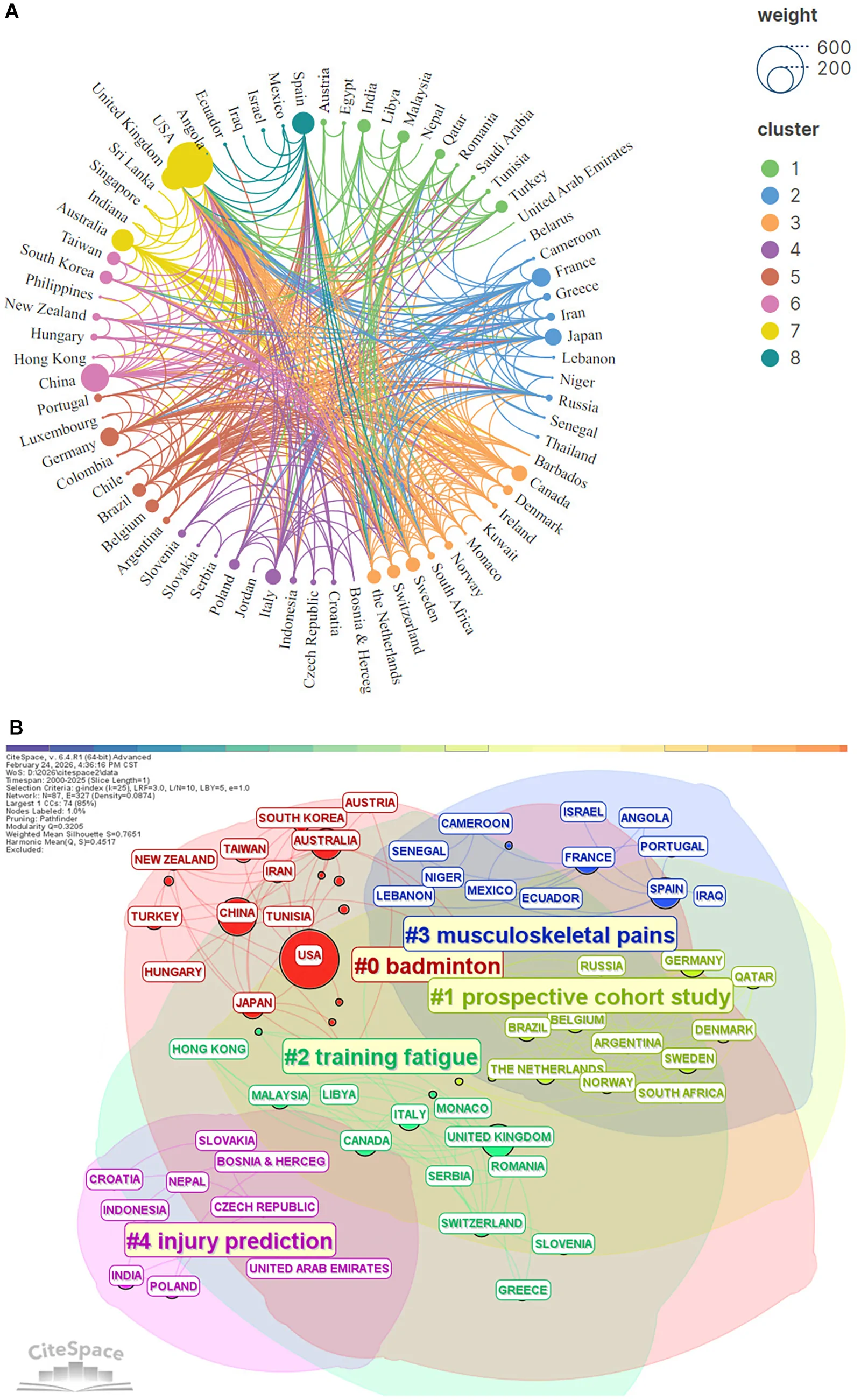
Country/region cooperation network for publications on racket sport-related injuries (2000−2025). **(A)** Cooperative relationships. **(B)** Country/region cooperation research topics.

The CiteSpace cluster analysis (Modularity Q = 0.3205, Silhouette S = 0.7651) systematically categorized the main international cooperation research topics into five categories (Figure 3B): (1) badminton injuries were predominant among countries such as the USA, China, Hungary, and Japan; (2) prospective cohort studies were predominant among countries such as Germany, Brazil, Denmark, and Belgium; (3) training fatigue was predominant among countries such as Italy, the United Kingdom, Canada, Greece, and Switzerland; (4) musculoskeletal pain was predominant among countries such as France, Spain, Cameroon, Mexico, and Portugal; and (5) injury prediction was predominant among countries such as Poland, India, Indonesia, Croatia, and the Czech Republic.

#### 3.3.2 Core institutions and cooperation networks

Core research institutions were mainly concentrated in the sports science and medical fields. The top five institutions in terms of publication volume were the Mayo Clinic (USA, 34 articles), University of Liege (Belgium, 34 articles), Harvard University (USA, 31 articles), Ningbo University (China, 28 articles), University of Amsterdam (Netherlands, 28 articles), University of California System (USA, 27 articles), and Harvard University Medical Affiliates (USA, 25 articles). By type, universities (73.7%), hospitals (11.8%), and sports medicine institutions and research centers (8.0%) were the main research centers. The institutional cooperation network analysis revealed that domestic partnerships were highly concentrated at 62% (e.g., between Ningbo University and Beijing Sport University), whereas transnational collaborations remained sparse, with only 15.1% of published articles involving international cooperation. Furthermore, independently authored papers accounted for 22.9%.

Using VOSviewer, we created a network collaboration diagram for institutions that occurred in the literature more than five times, with a point centrality of 1.18 and total link strength of 1.34. Thus, we established a collaborative network of ten core institutions in research on racket sport-related injuries (Figure 4), comprising the following clusters: cluster 1 (red): Stanford University, Ohio State University, University of Pittsburgh, University of Pennsylvania, and Texas State University, with a main research focus on musculoskeletal injuries in tennis; cluster 2 (green): Loughborough University, Shanghai University of Sport, University of Granada, University of Malaya, and University of Murcia, with a main research focus on badminton injury epidemiology; cluster 3 (dark blue): Monash University, Tennis Australia, University of Queensland, and Victoria University, with a main research focus on magnetic resonance imaging in sports injury assessment for tennis; cluster 4 (yellow): Amer Sports Medicine Institute, Scottsdale Sports Clinic, University of Copenhagen, University of Ghent, and US Tennis Association, with a main research focus on sport-specific adaptations in overhead athletes; cluster 5 (purple): University of Lyon, Aix Marseille University, University of Montreal, and University of Exeter, with a main research focus on biomechanical research in tennis; cluster 6 (light blue): Karolinska Institute, Sophiahemmet University, University of Tokyo, and Huazhong University of Science and Technology, with a main research focus on athletic injuries in tennis players and badminton players; cluster 7 (orange): Mayo Clinic, National Cheng Kung University, Tulane University, University of Sydney, and University of Western Australia, with a main research focus on injuries and rehabilitation in racket sports; cluster 8 (brown): Academic Center for Evidence-based Sports medicine (ACES), Royal Netherlands Lawn Tennis Association, University of Amsterdam, and Vrije University Amsterdam, with a main research focus on injury prevention in racket sports; cluster 9 (pink): Ningbo University, Beijing Sport University, Hong Kong Polytech University, and University of Auckland, with a main research focus on lower-limb injuries in racket sports; and cluster 10 (Light orange): University of Calgary, International Olympic Committee, University of Nottingham, and University of Oslo, with a main research focus on athletic injuries during the Olympic Games.

**Figure 4:**
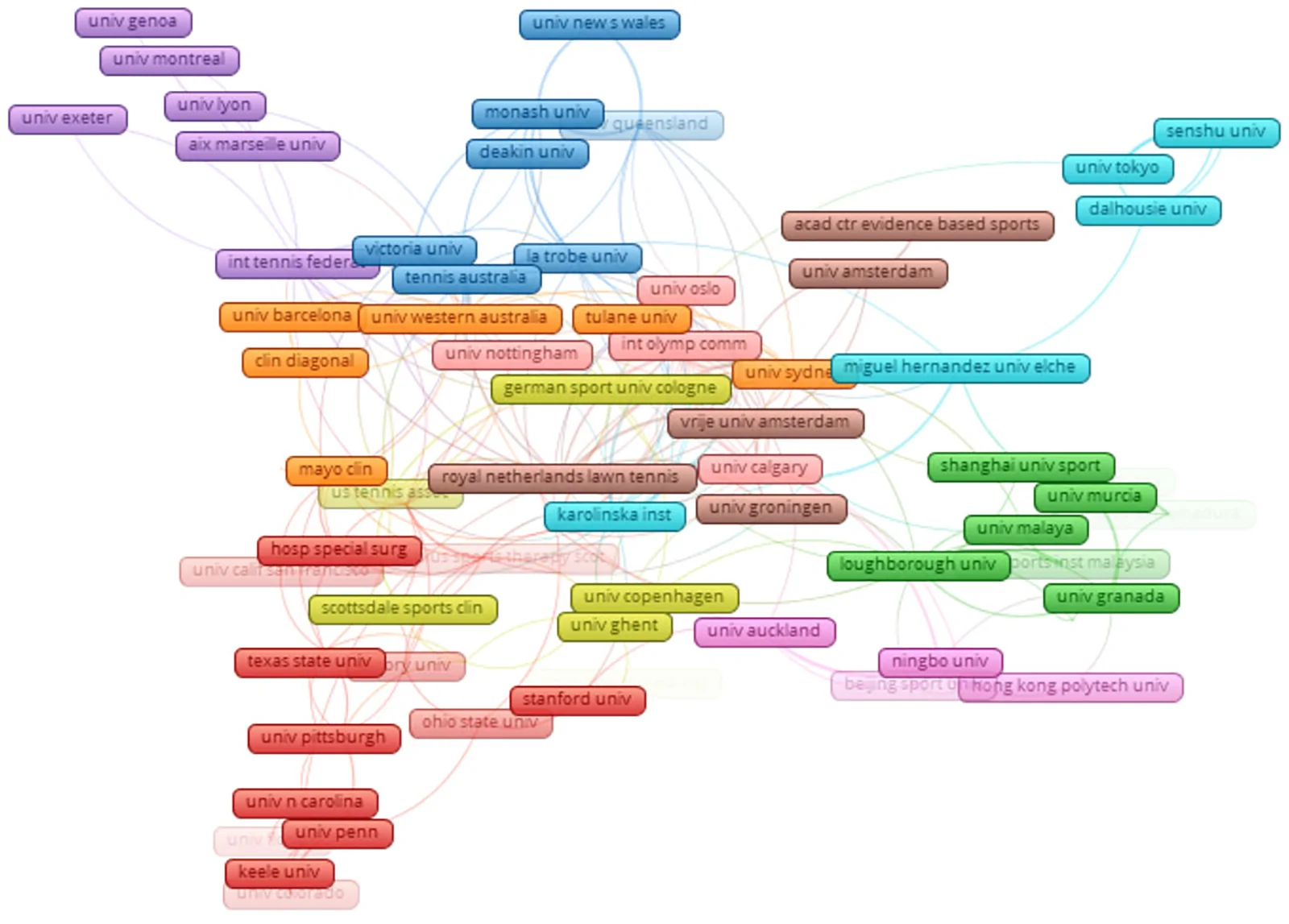
Core institutions cooperation networks for publications on racket sport-related injuries (2000−2025).

### 3.4 Core authorship and cooperation networks

According to Price's Law, 130 core authors were identified. The top five authors by publication volume were Ellenbecker, Todd S. (USA, 27 articles, focusing on tennis injury epidemiology, pathophysiology, and treatment); Pluim, Babette M. (Netherlands, 21 articles, focusing on tennis injury rates and injury prevention); Reid, Machar M. (Australia, 20 articles, specializing in impact of serve types on lumbar kinematics); Gu, Yaodong (China, 20 articles, focusing on lower-limb biomechanics in badminton players); Cools, Ann M. (Belgium, 15 articles, researching overuse injury); and Fernandez-Fernandez, Jaime (Spain, 14 articles, researching sports injuries in young athletes).

Using VOSviewer, we created a network collaboration diagram for authors who occurred more than five times in the literature, with a point centrality of 1.11 and a total link strength of 1.27. The author collaboration network formed six clusters (Figure 5), as follows: cluster 1 (red): core authors comprising Pluim, Babette M.; Bahr, Roald; and Casals, Marti, with the main research topics of sports injury and illness incidence at the Olympic Games; cluster 2 (green): core authors comprising Kulpa, Richard; Bideau, Benoit; and Martin, Caroline, with the main research topics of biomechanics of sport injuries and potential injury risks in tennis players; cluster 3 (blue): core authors comprising Fernandez-Fernandez, Jaime; Moreno-Perez, Victor; and Sanz-Rivas, David, with the main research topic of epidemiology of musculoskeletal injuries in tennis players (focusing on characteristics such as age, gender, and bodily injury); cluster 4 (yellow): core authors comprising Reid, Machar M.; Elliott, Bruce C.; and Rogowski, Isabelle, with the main research topic of upper limb joint muscle/tendon injury in tennis players; cluster 5 (purple): core authors comprising Ellenbecker, Todd S.; Ben Kibler, W.; and Roetert, E.P., with the main research topics: joint rotational strength and range of motion in elite tennis players (especially young female athletes); and cluster 6 (light blue): core authors comprising Johansson, Fredrik R.; Cools, Ann M.; and Skillgate, Eva, with the main research topic of training load and its association with sports injury in competitive adolescent tennis players (results from the SMASH cohort study).

**Figure 5:**
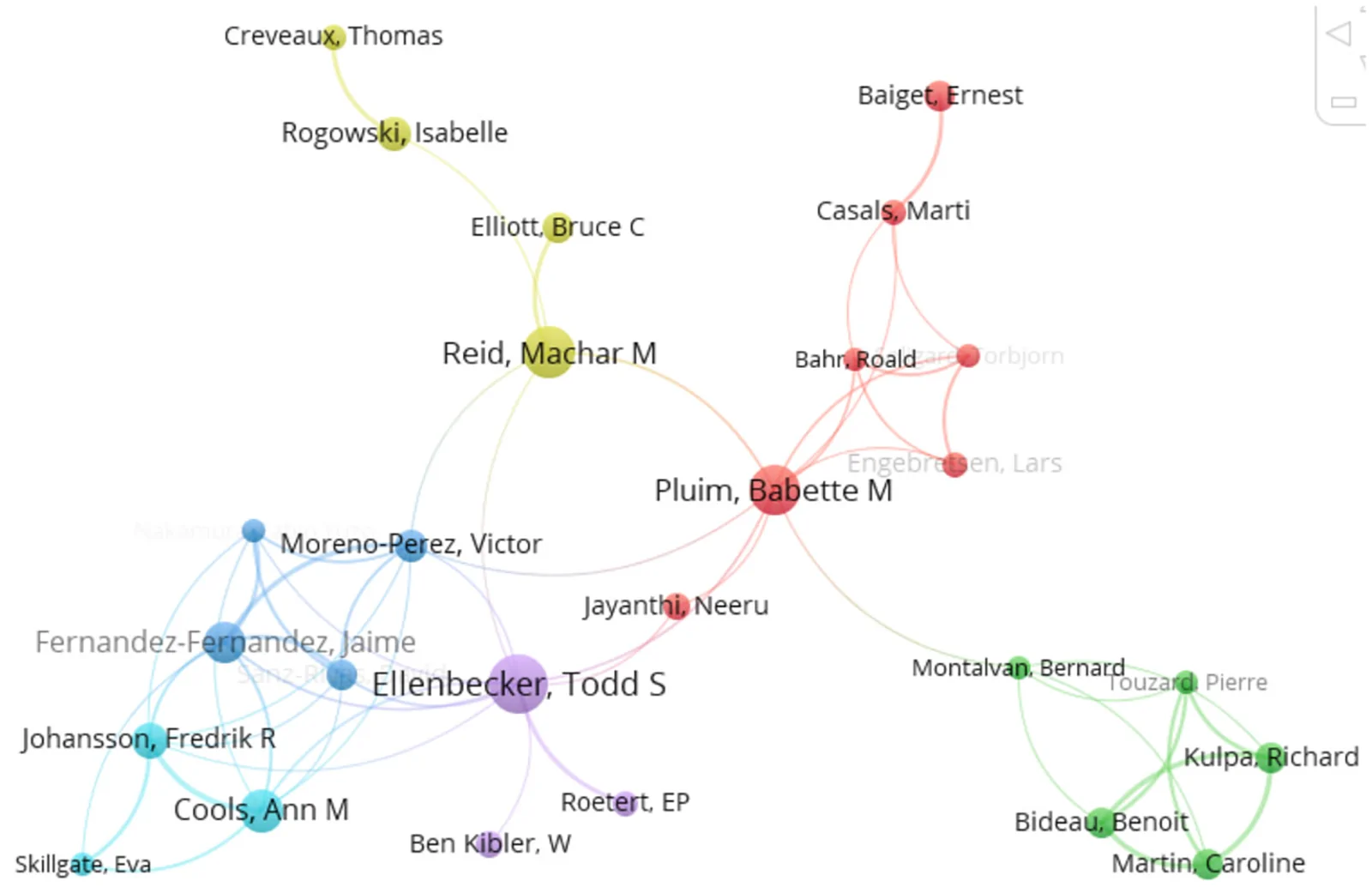
Core authors cooperation networks for publications on racket sport-related injuries (2000−2025).

### 3.5 Keyword co-occurrence and research hotspots

#### 3.5.1 Keyword frequency analysis

The top 10 keywords in terms of frequency were tennis (525 times), badminton (254 times), injury prevention (168 times), athletic injuries (166 times), adolescents (137 times), epidemiology (124 times), tennis elbow (99 times), biomechanics (80 times), MRI (67 times), and musculoskeletal injuries (63 times). These keywords cover core research content, including sport participant age, sport type, injury location and type, mechanism, diagnosis, and prevention.

#### 3.5.2 Cluster analysis of research hotspots

Analyzing the frequency and distribution of keywords facilitates the identification of trending research topics and their dynamic evolutionary trends (50). Figure 6 presents the cluster analysis of all keywords extracted from the included publications. The clusters exhibit distinct boundaries and strong internal linkages, demonstrating the diversity of research topics in this field. The CiteSpace cluster analysis (Modularity Q = 0.5743, Silhouette S = 0.8343) systematically categorized the research hotspots of racket sport-related injuries into 13 core clusters. The favorable Q and S values indicate significant and robust clustering results, with well-structured cluster partitioning and high internal consistency within each cluster (51, 52). Accordingly, the 13 core clusters formed six interconnected research domains, including epidemiological characteristics, sport-specific injury features, chronic overuse injuries, acute traumatic injuries, imaging-based injury diagnosis, and medical injury intervention. The detailed classification and research focus of each cluster are elaborated as follows.

**Figure 6:**
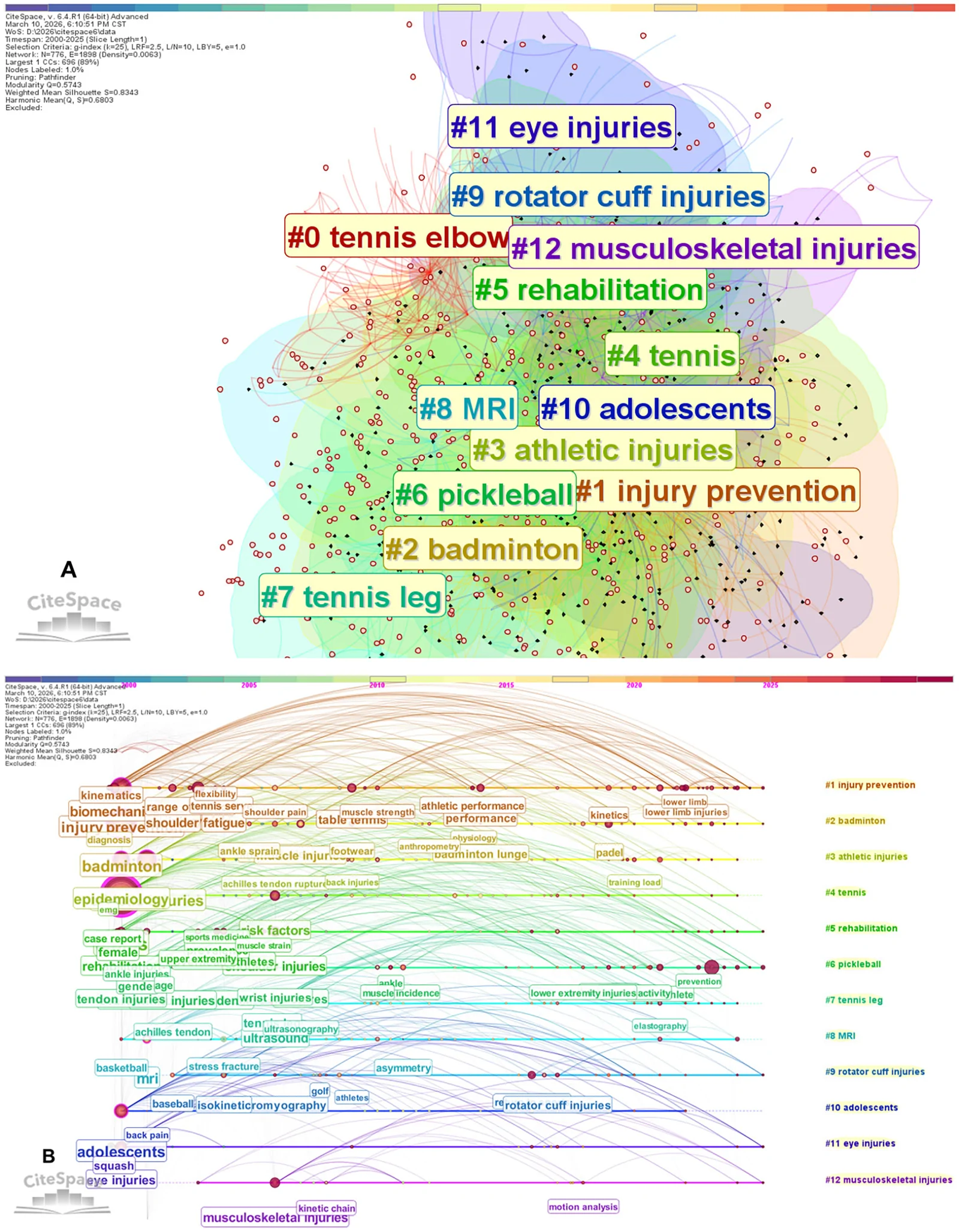
Keyword clusters for publications on racket sport-related injuries (2000−2025). **(A)** Keyword clusters of publications on racket sport-related injuries. **(B)** Keyword timeline of publications on racket sport-related injuries.

##### 3.5.2.1 Epidemiological characteristics and injury prevention (clusters 1, 3, and 10)

This theme focuses on the epidemiological characteristics of racket sport-related injuries, covering injury risk factors, population distribution, and adolescent-specific injury prevention. Cluster 1 centers on multiple risk factors associated with racket sport-related injuries, which are categorized into the following three dimensions: subjective factors, including athletes’ physical condition and athletic performance; objective factors, referring to sports venue environments; and training factors, encompassing range of motion, serving technique, plyometric training, training intensity, and training duration. For example, in tennis training, wearable devices enable data-driven coaching through shot recognition and workload monitoring, thereby enhancing training effectiveness and reducing the incidence of injuries (53). Cluster 3 examines the population distribution characteristics of racket sport-related injuries. Relevant research covers injury incidence; demographic characteristics such as age and gender; and risk factors related to athletic status, technical proficiency, training load, and sports equipment. Cluster 10 explores injury characteristics and targeted prevention strategies for adolescent racket sport participants, fully considering their unique physical growth and developmental features (54).

##### 3.5.2.2 Racket sport-specific injuries (clusters 2, 4, and 6)

This theme centers on sport-specific injuries occurring in badminton, tennis, and pickleball, with distinct injury mechanisms and pathological characteristics across sports. Cluster 2 encompasses the typical injury characteristics of badminton-related musculoskeletal damage, including injuries to the knees, muscles, and feet, as well as preventive strategies based on artificial intelligence-assisted movement pattern recognition and scientific training formulation (55). Cluster 4 explores tennis-specific injuries, particularly upper limb lesions induced by serving movements, and their underlying biomechanical mechanisms. Cluster 6 examines pickleball-related lower-limb injuries and corresponding risk factors. As a rapidly developing racket sport popular among older adults and amateur participants, pickleball exhibits unique injury patterns. Meanwhile, research on high-injury-risk sports has explored new technologies for injury prevention. For instance, wireless inertial sensors and neural networks have been applied to analyze badminton strokes, aiming to reduce injury incidence during play (56).

##### 3.5.2.3 Chronic overuse injuries (clusters 0, 7, and 9)

This topic addresses chronic overuse injuries, which constitute a core research direction in racket sport injury research. Cluster 0 investigates the clinical manifestations and standardized treatment modalities of tennis elbow, whose predominant symptoms include local inflammation and pain, such as epicondylitis, medial elbow pain, bicipital tendonitis, elbow osteoarthritis, and elbow stiffness. Common interventions consist of physiotherapy, dry needling, corticosteroid injections, extracorporeal shock wave therapy, and arthroscopic treatment (57). Cluster 7 focuses on the pathogenesis, clinical diagnosis, and functional rehabilitation of tennis leg. Cluster 9 analyzes rotator cuff injuries, covering diagnostic evaluation, surgical repair, and postoperative rehabilitation. Tennis elbow, tennis leg, and rotator cuff injuries are typical chronic overuse injuries attributed to repetitive overhead movements and cumulative mechanical loading in racket sports.

##### 3.5.2.4 Acute traumatic injuries (clusters 11 and 12)

This topic characterizes acute traumatic injuries in racket sports. Cluster 11 identifies eye injuries as a core acute injury type, which frequently occur among young athletes during racket sport participation. Relevant studies elaborate on injury characteristics and corresponding preventive approaches relying on professional protective eyewear (58). Cluster 12 describes musculoskeletal acute injuries sustained in competitions, routine training, and daily fitness activities. Such injuries result from abrupt mechanical loading on musculoskeletal tissues and primarily include muscle contusions, muscle tears, fractures, and joint dislocations.

##### 3.5.2.5 Imaging-based injury diagnosis (cluster 8)

This cluster investigates imaging-based injury diagnosis in racket sports. It addresses the application of MRI and imaging techniques in the clinical diagnosis of racket sport-related musculoskeletal injuries and tendinopathy (59).

##### 3.5.2.6 Rehabilitation of sports injuries (cluster 5)

This cluster elaborates on diverse intervention strategies for racket sports injuries, mainly encompassing physiotherapy, pharmacotherapy, and surgical treatment. Physiotherapy serves as a primary intervention for chronic overuse injuries, including extracorporeal shock wave therapy and systematic rehabilitation training (23). By contrast, surgical intervention is generally reserved for severe acute injuries, such as tendon rupture.

#### 3.5.3 Evolution of research frontiers

As shown in Figure 7, keyword time-zone and burst analysis intuitively demonstrate the dynamic evolutionary trends of research hotspots in the field of racket sport-related injuries, which can be divided into three distinct and progressive developmental stages with independent research focuses.

**Figure 7:**
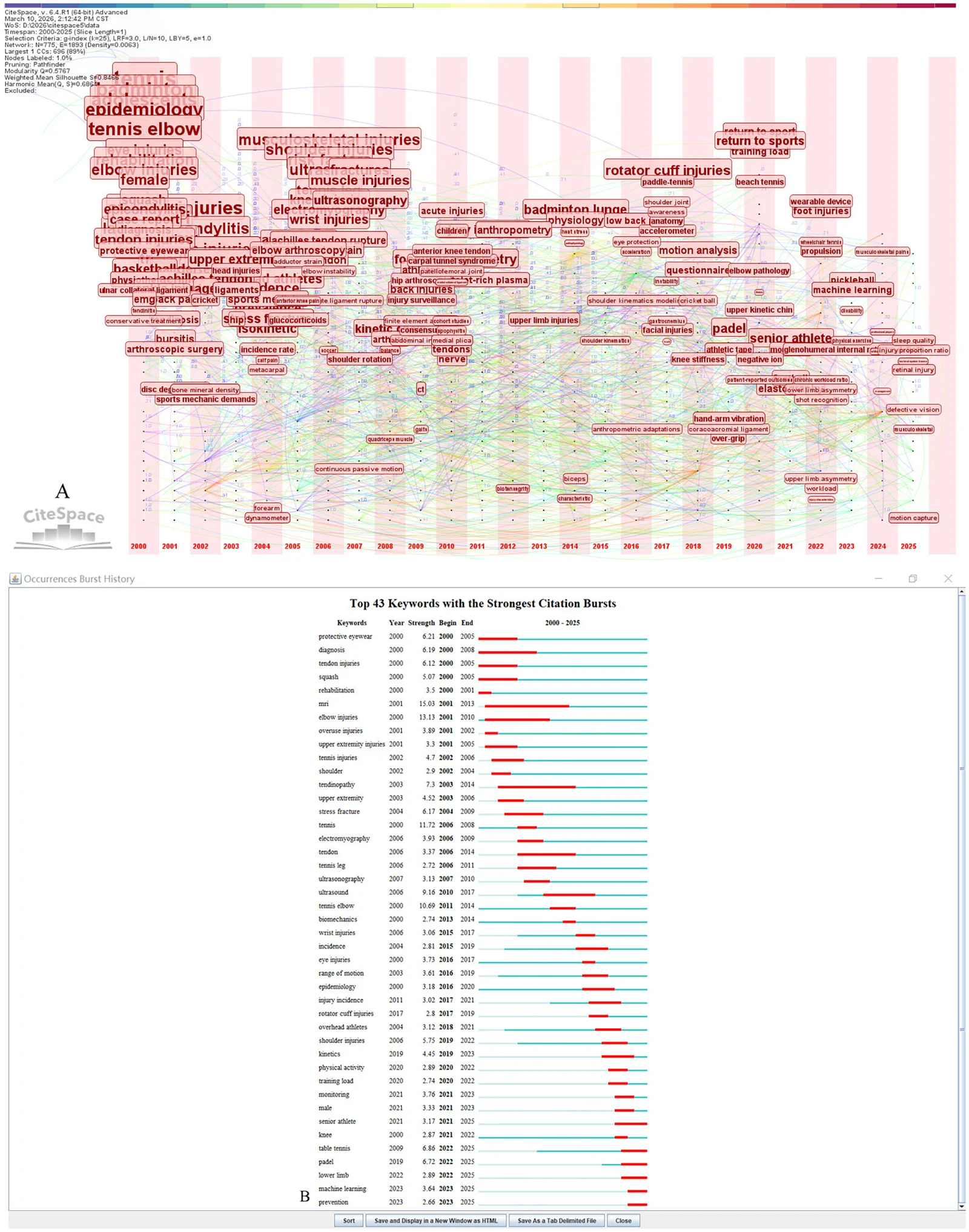
Evolution of research on racket sport-related injuries (2000−2025). **(A)** Keyword time-zone analysis of publications on racket sport-related injuries. **(B)** Keyword burst analysis of publications on racket sport-related injuries.

**Stage 1 (2000–2010): Epidemiological investigation and traditional treatment stage.** The research in this first decade centered largely on macroscopic epidemiological investigations and standard clinical management of injuries associated with racket sports. Studies focused on traditional racket disciplines such as tennis, badminton, and squash. Prevailing themes included injury incidence, demographic profiles of female athletes, injury surveillance systems, conventional rehabilitation, arthroscopic surgery, and protective gear improvement (60). This stage laid a basic theoretical foundation for understanding the prevalence patterns and primary treatment strategies of these injuries.

**Stage 2 (2010–2020): Biomechanical mechanism and standardized intervention stage.** This decade witnessed a clear research shift from macroscopic statistical description to microscopic mechanism analysis and standardized rehabilitation intervention. Research priorities centered on exploring the biomechanical pathogenesis of sports injuries, summarizing diversified physical therapy modalities, and optimizing targeted rehabilitation technologies (61, 62). In particular, extracorporeal shock wave therapy gradually became a mainstream auxiliary treatment for chronic racket sport-related injuries, which effectively improved the comprehensiveness and standardization of clinical injury intervention in this field (63).​

**Stage 3 (2021–): Intelligent technology and precision prevention stage.** In the latest stage, interdisciplinary intelligent technologies have driven the research frontier toward precise and individualized injury prevention. Emerging research hotspots represented by artificial intelligence, machine learning, wearable devices, motion capture, and artificial neural networks have been widely applied in sports injury research (28, 64). Wearable sensing technology allows for real-time monitoring of muscle activity and joint loading, while intelligent algorithms support the construction of accurate injury risk prediction models. The MediaPipe-based deep learning tool estimates joint angles and motion patterns in pickleball, offering an approximate alternative to visual coaching analysis and enabling rapid monitoring of kinematic parameters to identify areas for improvement (65).

### 3.6 Distribution of publishing journals and category

A total of 571 journals have contributed to publications on racket sports-related injuries. The top 10 most-productive journals are listed in Table 2. The *Brit J Sport Med* yielded the largest number of publications, representing the core journal source in this research field.

**Table 2:** Top 10 journals by number of publications on racket sport-related injuries (2000−2025).

Rank	Journal abbreviation	Number of publications	Journal impact factor	Journal impact factor quartile
1	*Brit J Sport Med*	101	16.2	Q1
2	*Clin Sport Med*	43	1.6	Q3
3	*Am J Sport Med*	39	4.5	Q1
4	*Orthop J Sports Med*	29	2.5	Q2
5	*PLoS One*	28	2.6	Q2
5	*Rev Bras Med Esporte*	28	0.652	Q4
6	*J Sport Med Phys Fit*	27	1.3	Q3
6	*J Sport Sci*	27	2.5	Q2
7	*Phys Ther Sport*	26	2.4	Q1
8	*Clin J Sport Med*	25	1.8	Q2
9	*Int J Env Res Pub He*	24	N/A	N/A
9	*Physician Sportsmed*	24	1.8	Q2
10	*J Sci Med Sport*	22	3.4	Q1

Journal impact factor from JCR 2025.

We mapped the distribution of journals related to our research topic, which includes sports medicine, sports injuries, rehabilitation treatment, and injury prevention. Since our research topic differs from the Journal Citation Reports (JCR) journal subject classification, mapping processing was required. First, 571 journals were categorized into 254 subject areas based on the JCR. Then, a subject classification map was created by associating these with the following four aspects of the research topic: sports medicine, sports injuries, rehabilitation therapy, and injury prevention. Subsequently, the subject categories of journals within this thematic area were counted. The statistical results are presented in Table 3.

**Table 3:** Categorization of publications on racket sport-related injuries (2000–2025).

Rank	Journal category	Number of publications	Rank	Journal category	Number of publications
1	Sport Medicine	470	16	Anthropology	3
2	**Rehabilitation**	425	17	Biology	3
3	Sport Sciences	343	18	Education, Scientific Disciplines	3
4	Medicine, General and Internal	155	19	Materials Science	3
5	**Sport Injury**	110	20	Nursing	3
6	**Injury Prevention**	81	21	Psychiatry	3
7	Multidisciplinary Sciences	79	22	Social Sciences	3
8	Engineering	53	23	Food Science and Technology	2
9	Ophthalmology	38	24	Psychology	2
10	Rheumatology	13	25	Sport Injury and Rehabilitation	2
11	Computer Science	10	26	Behavioral Sciences	1
12	Pediatrics	10	27	Communication	1
13	Neurosciences	9	28	Economics	1
14	Dermatology	4	29	Hematology	1
15	Integrative and Complementary Medicine	4	30	Mathematics	1

**Principles for subject-classification mapping:** (**i**) If the journal involved specific body parts such as hands, feet, muscles, and ligaments, we mapped it to sports injuries; (**ii**) if the journal involved orthopedics, surgery, drugs, or treatment, we mapped it to injury rehabilitation; (**iii**) if the journal involved public health, environmental and occupational health, or epidemiology, we mapped it to injury prevention; (**iv**) if the journal involved specific kinesiology, exercise, physiology, or sports science terminology, we mapped it to sports science; (**v**) if the journal involved medicine and kinesiology, we mapped it to sports medicine; (**vi**) if the journal involved psychology, sociology, engineering, computer science, or other disciplines, we retained the original discipline classification without mapping.

We counted the number of papers published in journals focusing on rehabilitation, sports injuries, and injury prevention. As Table 3 shows, there were 425 journal articles on rehabilitation, 110 on sports injuries, and 81 on injury prevention. A chi-squared goodness-of-fit test was conducted to determine whether the distribution of the articles across the three topics deviated significantly from a uniform distribution. There were extremely significant differences in article count across the three research themes (*χ*^2^ = 354.55, *p* < 0.001, Cramér's V = 0.54). The rehabilitation topic dominated the dataset with 69.0% of all articles, followed by the sports injuries (17.9%) and injury prevention (13.1%) topics. Pairwise comparisons confirmed that the rehabilitation topic was significantly different from both other categories, while the sports injuries and injury prevention topics did not differ significantly from each other. After the Bonferroni correction (*α*’ = 0.0167), rehabilitation differed significantly from both the sports injuries and injury prevention topics. However, sports injuries and injury prevention do not differ significantly from each other (*p* = 0.036 > 0.0167). The large effect size (Cramér's V = 0.54) indicates that the differences in article counts across the three research topics were not only statistically significant but also practically meaningful.

### 3.7 Highly cited papers analysis

A total of three highly cited papers in the field of racket sports and sports injuries were retrieved from the Web of Science Core Collection. According to the Essential Science Indicators (ESI) criteria, highly cited papers refer to publications ranked in the top 1% of citations in the field of clinical medicine within their corresponding publication years, based on the data updated as of November–December 2025. The three representative highly cited papers are summarized as follows: (1) The 2016 consensus statement entitled *How much is too much? (Part 1) International Olympic Committee consensus statement on load in sport and risk of injury* [Soligard et al., 2016, 726 citations (66)] systematically explains the correlation between training load and sport-related injury risk and provides practical load-management guidelines for athletes, coaches, and supporting staff; (2) The 10-year epidemiological study *Epidemiology of athletic knee injuries: A 10-year study* [Majewski et al., 2006, 644 citations (60)] retrospectively analyzed 6,434 patients with 7,769 sports-related knee injuries. The findings confirmed that lateral collateral ligament injuries and medial meniscus injuries are closely associated with tennis participation; (3) The prospective Olympic Games study *Sports injury and illness incidence in the Rio de Janeiro 2016 Olympic Summer Games: A prospective study of 11274 athletes from 207 countries* [Soligard et al., 2017, 323 citations (67)] systematically recorded the incidence of sports injuries and illnesses through dual-source monitoring, including daily reports from all National Olympic Committee medical teams and on-site medical records from Rio 2016 polyclinics and designated medical venues. Statistical results indicated that 8% of participating athletes sustained at least one sports injury and 5% suffered from illnesses during the competitions, which was slightly lower than the injury and illness rates reported in the 2008 and 2012 Summer Olympic Games.

## 4 Discussion

### 4.1 Trends in the growth of academic papers

In comparison with 2000–2019, the 2020–2025 phase presented a substantially higher CAGR and greater publication-growth magnitude, accompanied by greatly improved growth stability. This remarkable growth can be attributed to several interrelated factors. To begin with, rising global participation in racket sports has accumulated rich clinical data, while research partnerships between universities, sports teams, and rehabilitation hospitals have kept expanding (3). Secondly, the global racket sports market was estimated at USD 26.81 billion in 2025 and is expected to reach USD 43.59 billion by 2033, with a CAGR of 5.55% across the forecast period (68). Thirdly, the temporal keyword burst analysis in the present study confirmed that the number of published papers on racket sport-related injuries increased accordingly with the development of innovative technologies such as artificial intelligence and wearable devices. Moreover, the popularization of wearable sensors, motion capture, and AI-based biomechanical modeling has reduced technical obstacles for quantitative injury research and supported applied empirical work. Collectively, these multiple driving factors will sustain innovative progress in research on racket sports injuries.

### 4.2 Cross-country/regional collaboration characteristics

Bibliometric analysis of geographical distribution and research collaboration revealed the following characteristics of racket-sport-related injuries.

#### 4.2.1 Research output was unevenly distributed and closely linked to economic development

The publication volume on racket sport-related injuries revealed significant cross-national imbalances. The top 10 productive economies contributed 64.5% of all papers, with developed Western countries, led by the United States (accounting for 22.5%), dominating the field. Spearman’s correlation analysis confirmed a significant positive correlation between national research productivity and per capita GDP (*ρ*=0.538, *p* < 0.001), indicating that favorable economic conditions support sustained investment in sports medicine clinical data collection, biomechanical testing, and long-term cohort research. While China ranks second in total publications, its per capita GDP lags far behind its European and North American counterparts, suggesting that its research output was largely dependent on its large population base rather than equal per capita research investment.

#### 4.2.2 Fragmented international cooperative clusters with fixed regional partnerships were identified

Eight geographically bounded cooperative clusters (Figure 3A) demonstrated regionalized closed collaboration rather than globalized open exchange. The U.S.-centered cluster maintained tight links with Australia, the UK, and Singapore, forming the most mature transnational research alliance. European scholars formed multiple isolated small groups, including France–Japan–Russia, Italy–Central Europe, Germany–South America, and Spain–North Africa. China's cooperative scope mainly covered the East Asian and Oceania regions (South Korea, New Zealand, Taiwan, and Hungary), with limited connections to the North American core teams.

Such regional segmentation restricts cross-country knowledge exchange. In terms of collaborative research themes (Figure 3B), each cluster maintained relatively independent research priorities: North American teams focused on badminton injury observation and prospective cohort design; European groups concentrated on musculoskeletal pain and training fatigue; and East Asian and Central European scholars prioritized adolescent injury prediction. This thematic divergence results in fragmented research systems that lack unified global research frameworks.

### 4.3 Institutional collaboration characteristics

Research institutions engaged in this field were predominantly universities, with hospitals offering auxiliary clinical support. Universities contributed 73.7% of all publications and served as core platforms for basic biomechanical and epidemiological investigations. Hospitals (11.8%) and professional sports medicine centers (8.0%) supplied clinical injury data and rehabilitation verification, collectively constituting a two-pillar research system of “universities plus clinical institutions.” High-productivity institutions were distributed across multiple regions, including North American medical centers (e.g., Mayo Clinic, Harvard University) and European sports medicine platforms in Belgium. In China, Ningbo University and Beijing Sport University represent the leading research institutions in this domain.

The collaboration network displayed a strong preference for domestic cooperation, whereas cross-institutional communication across national borders remained inadequate. Institutional collaboration data revealed obvious localization features: 62% of collaborative publications originated from domestic institutional partnerships (e.g., Ningbo University and Beijing Sport University). Single-institution independent studies accounted for 22.9%, and merely 15.1% of publications involved cross-border institutional cooperation. The institutional collaboration network (Figure 4) formed 10 discipline-oriented clusters with relatively closed intra-cluster cooperation. Specifically, medical institutions in the United States focused on tennis-related musculoskeletal injuries; universities in the United Kingdom and Southeast Asia prioritized epidemiological research on badminton injuries; Nordic institutions concentrated on injury monitoring for Olympic sports; and Chinese sport universities mainly conducted research on lower-limb biomechanics in racket sports. Limited cross-border academic links existed between these clusters, which restricted international exchange of research findings.

### 4.4 Core author collaboration characteristics

The core author teams remained stable, with each confined to distinct research domains. According to Price's Law, 130 core authors were screened, exhibiting distinct specialized research directions with minimal thematic overlap, as follows: Todd S. Ellenbecker and Babette M. Pluim formed the global leading team focusing on tennis injury epidemiology and prevention; Australian scholar Machar M. Reid specialized in tennis serving biomechanics; Chinese scholar Gu Yaodong focused on badminton lower-limb biomechanics; and European researchers led in adolescent overuse injury research. This clear niche differentiation avoids repetitive research.

The author cooperative network was divided into six mutually independent clusters (Figure 5), with limited cross-group communication. Each cluster had fixed core members and exclusive research topics. Connections between different clusters were sparse, meaning research findings in each cluster were rarely integrated into unified prevention schemes.

Overall, in the field of racket sport-related injuries, national research output was unevenly distributed and closely related to economic development levels. Fragmented international collaboration clusters with fixed regional partnerships were identified, along with insufficient cross-border institutional and author collaborations.

### 4.5 Research hotspots

After synthesizing the six research domains, the hotspots of racket sport-related injuries presented with three distinct traits. First, studies focused on specific types of injury: chronic overuse injuries and acute trauma. Second, the field followed a complete research logic, progressing from population-based epidemiological investigations to sport-specific mechanism exploration, imaging-based objective diagnosis, and clinical intervention and rehabilitation, thereby forming a closed-loop research system. Third, emerging hotspots centered on adolescents, AI-assisted motion analysis, and intelligent imaging diagnosis have continuously expanded in recent years, driving the field toward a future focused on youth, technology-enabled solutions, and precise prevention-oriented approaches.

To further advance racket sport injury research, scholars need to shift from small-scale, single-sport, single-discipline independent explorations to large-sample, cross-national collaborative research. Future research should integrate intelligent monitoring technology and population cohort studies. By breaking down geographical, institutional, and thematic barriers through global collaborative networks, these efforts will enable the formation of systematic, generalizable theoretical systems for injury prevention, diagnosis, and rehabilitation.

### 4.6 Research evolution

Keyword time-zone and burst analyses divided racket sport injury research into three progressive stages. From 2000 to 2010, studies focused on epidemiological description and conventional clinical treatment, establishing basic data. The 2010–2020 phase shifted to biomechanical pathogenesis and standardized rehabilitation interventions for chronic overuse injuries. Since 2021, intelligent monitoring and machine learning have driven research toward proactive, personalized injury prevention. Overall, the field has shifted from passive post-injury treatment to active risk prediction, with methodologies evolving from basic statistics to quantitative intelligent measurement and research covering more sports and populations.

### 4.7 Research gaps

Despite the expanding research on racket sport-related injuries, several key research gaps persist in this field. First, the research population is structurally unbalanced. Existing studies predominantly focused on elite competitive athletes, with limited attention paid to recreational participants. Second, sport-specific epidemiological data and large-scale cohort studies across various racket sports remain inadequate, restricting comprehensive insight into distinct injury characteristics in different sports and populations. Second, insufficient cross-border and cross-institutional cooperation hinders the development of multi-center, large-sample studies, limiting the translation and generalization of research findings and lowering the overall quality of the domain. Third, current research methodologies are relatively narrow, dominated by cross-sectional surveys and clinical observations. High-quality evidence from long-term follow-up studies, randomized controlled trials, and systematic reviews is scarce, resulting in a low level of evidence-based practice for racket sport injury prevention and treatment. Fourth, innovative research on artificial intelligence, wearable devices, and personalized rehabilitation is still in the initial stages, lacking mature technical systems and clinical verification, which restricts its practical application in precise injury intervention. Finally, emerging racket sports including pickleball and air badminton are developing rapidly, and the corresponding injury prevention measures cannot keep up with the growing number of participants in these sports.

### 4.8 Research limitations

This study has several limitations. First, data were retrieved from three databases (WOS, Scopus, and PubMed), meaning relevant records indexed in other platforms such as SPORTDiscus and Rehabilitation & Sports Medicine Source may be omitted, which undermines the comprehensiveness of the analysis; these databases were not incorporated due to access constraints. Second, only English-language publications were included to facilitate standardized data cleaning and software processing, which may introduce bias in the geographical distribution of research output (Table 3) and lead to incomplete global data coverage. Third, some critical datasets, including the annual number of participants in racket sports and national-level incidence data of associated sports injuries, are not freely available and require paid access. Restricted research funding prevented further analysis of additional indicators. Fourth, since the selected databases are all abstract databases, there is a time lag between their publication and that of the original papers. Consequently, delays in updating certain journals in 2025 will result in the absence of some data. Fifth, due to our inability to access data such as Olympic results, World Championship medals, or world rankings in our current research, we were unable to investigate the relationship between publishing activities and sports achievements, nor to gain deep insights into the mutual interactions between scientific capabilities and sports performance. We recommend this as a promising direction for future research. Finally, constrained by the limitations of literature visualization software, which lacks the capability for simultaneous multilingual-literature analysis, a comprehensive assessment of literature from Chinese databases (e.g., CNKI and Wanfang) could not be performed.

## 5 Conclusion

This comprehensive bibliometric analysis of the racket sport injury literature (2000–2025) found that publications have surged since 2020, and relevant studies were mainly distributed in sports medicine and orthopedic journals with insufficient interdisciplinary integration. National, institutional, and author collaboration networks suffered from unequal research resources, regional segregation, and disciplinary fragmentation. Keyword clustering divided the research into six core domains, which were dominated by chronic overuse injuries, with adolescent protection and intelligent monitoring as emerging hotspots. Research themes have evolved sequentially from epidemiological description to biomechanical exploration and intelligent precise prevention, shifting the core logic from passive injury treatment to proactive risk control. Three highly cited papers according to ESI proved that large-sample, standardized, and translational epidemiological and training-load research receives the greatest academic recognition. To advance this field, scholars need to strengthen transnational interdisciplinary cooperation; expand multi-population and cross-sport comparative research; adopt unified injury surveillance and quantitative load management frameworks; and construct systematic, generalizable prevention and rehabilitation systems for racket sport participants.

## Data Availability

The datasets presented in this study can be found in online repositories. The names of the repository/repositories and accession number(s) can be found in the article/Supplementary Material.
